# Production and purification of VP2 protein of porcine parvovirus expressed in an insect-baculovirus cell system

**DOI:** 10.1186/1743-422X-7-366

**Published:** 2010-12-10

**Authors:** Hongchao Zhou, Guizhe Yao, Shangjin Cui

**Affiliations:** 1 College of Veterinary Medicine, Northwest A&F University, Yangling, Shaanxi, 712100, China; 2 Division of Swine Infectious Disease, State Key Laboratory of Veterinary Biotechnology, Harbin Veterinary Research Institute of CAAS, Harbin, China 150001

## Abstract

The porcine parvovirus (PPV) VP2 protein was expressed in an insect-baculovirus cell system and was purified using Ni-NTA affinity column chromatography. The recombinant 6-His-tagged VP2 protein with molecular mass (Mr) of about 64 kDa was detected by anti-his antibody and anti-PPV serum. Electron microscopy showed that the purified VP2 protein assembled into spherical particles with diameters ranging from 20 to 22 nm. The expressed VP2 was antigenically similar to the native capsid protein according to HA and a Western blotting assay performed with polyclonal antibodies collected from an outbreak of PPV in one farm. This study provides a foundation for the application of VP2 protein in the clinical diagnosis of PPV or in the vaccination against PPV in the future.

## 1. Introduction

Porcine parvovirus (PPV) causes reproductive failure in pregnant sows. The infection occurs without clinical symptoms in adults; however, the virus can cross the placental barrier during the infection and cause the death of the fetuses, stillbirths and return to estrus [1]. More recently, PPV has gained importance as an agent able to increase the effects of porcine circovirus type 2 infection in the clinical course of postweaning multisystemic wasting syndrome (PMWS) [2,3], which is a significant disease in global swine production [4]. Widespread vaccination has been proposed as a cost-effective method to reduce the economical losses due to the endemic and worldwide prevalence of this virus [5,6].

PPV is a small, non-enveloped, single-stranded, negative-sense DNA virus. Capsids of PPV are assembled from three viral proteins (VP1, VP2, and VP3). The major structural protein, VP2 is the main target for neutralizing antibodies in PPV [7,8]. When VP2 was expressed in large amounts using the baculovirus expression vector system, it assembled into virus-like particles (VLPs) similar in size and morphology to the original virions [7]. PPV VP2 VLPs induced antibodies against PPV in immunised pigs [7] and rabbits [9]. PPV-cell or tissue-tropism determinants, host-range determinants, and determinants that confer hemagglutination properties have all been shown to be located in the capsid proteins [10-12]. It is noted that PPV VP2 was expressed by Lu et al. (2002) in pFastBac I and by Si et al. (2006) in pFastBacDUAL using insect cell-baculovirus systems and both groups demonstrated a 64 kDa band by Western-blot analysis.

Because VP2 is the main structural protein of PPV and constitutes most of the viral capsid, VP2 produced *in vitro *can self assemble into virus-like particles [13]. PPV VP2 VLPs exhibited positive immunoreactivity for PPV in a commercial ELISA [14]. Rueda et al. (2000) showed that contaminant baculovirus could be inactivated in preparations of PPV VP2 VLPs while retaining physical and immunological properties. VP2 VLPs have been produced and purified using a specific affinity Immobilized Metal Affinity Chromatography (IMAC) system for other parvoviruses such as B19 [15] and for infectious bursal disease virus [16]. To facilitate the use of PPV VP2 for diagnosis and vaccination, the current study attempted to identify an improved procedure for producing VP2 *in vitro *and for purifying this fusion protein.

There are many advantages to the use of VLPs in vaccines and for diagnosis. Compared to inactivated virus, which is currently used in vaccines, VLPs do not require the propagation of infectious virus, there is no risk of virus transmission or infection, production levels are much higher, production is cost effective, and VLPs are generally stable. The authors have previously expressed PPV VP2 in E. coli using the plasmid pET-32a (+) [17]. VP2 expressed in bacteria had similar antigenicity to native PPV VP2, as determined by Western blot analysis using polyclonal antibodies from pigs vaccinated against PPV [17]. PPV VP2 expressed in bacteria appears to have good immunogenicity, this is better for using as vaccine than the use as diagnosis antigen, for the antibody for E. coli in sera effects the ELISA assay. This provide us a compelling reason for expressing PPV VP2 in a baculovirus system, although baculovirus expression systems are likely to be more costly than bacterial expression systems for producing viral proteins and may present difficulty in purifying expressed proteins from insect cell and baculovirus constituents. PPV VP2 has been expressed in insect cell-baculovirus systems by a number of other groups previously [7,9,14,15]. PPV VP2 expressed by baculovirus in sf9 cells produced VLPs [7,9,14,15]. PPV VP2 VLPs induced antibodies against PPV in immunised pigs [7]and rabbits [14]. PPV VP2 VLPs exhibited positive immunoreactivity for PPV in a commercial ELISA [15]. Rueda et al. [1] showed that contaminant baculovirus could be inactivated in preparations of PPV VP2 VLPs while retaining physical and immunological properties. It is noted that PPV VP2 was expressed by [15] in pFastBac I and by Si et al. [9] in pFastBacDUAL using insect cell-baculovirus systems and both groups demonstrated a 64 kDa band by Western-blot analysis. VLPs produced in baculovirus-infected insect cells could then be used as a vaccine or as a diagnostic agent to detect the antibody produced by PPV infection or vaccination [18]. Baculovirus-infected insect cells have been used to produce VP2 VLPs of infectious bursal disease virus [19], and parvoviruses including PPV [14] and B19 [15]. To further facilitate the use of PPV VP2 for diagnosis and vaccination, the goal of the current study was to find an improved procedure for expressing PPV VP2 *in vitro *and to purify the fusion protein.

## 2. Materials and methods

### 2.1. Cells, virus, and reagents

Strain 20-06 of PPV was isolated from a dead fetus delivered from a sow diagnosed with reproductive failure. HRP-labeled anti-pig serum was purchased from Sigma (St. Louis, Missouri, USA). Ni-NTA His Bind resin was obtained from Invitrogen (Carlsbad, California, USA). Prestained protein ladder was purchased from Fermentas International Inc. (Burlington, Canada). Swine anti-PPV serum samples, with serum neutralization titer, were obtained from the Harbin Veterinary Research Institute, CAAS. The serum samples were collected from an outbreak of PPV in 2008 in one farm in Heilongjiang Province.

### 2.2. Construction of recombinant plasmids and recombinant bacmid

Genomic DNA was extracted from the cell-cultured strain 20-06 of PPV by the classical phenol-chloroform extraction method and was used as a template to amplify the VP2 fragment by PCR. The PPV VP2 gene was amplified with the primers PPV-VP2 FD (TATGGATCCGATGAGTCATCATCACCATCACCATAGTGAAAATGTGGAACAAC) and PPV-VP2 RV (GCGTCGACTATGAGTTAGAGTTTGTATTAG). The underlined nucleotides represent BamHI and SalI restriction sites, respectively. The PCR products were digested with BamHI and SalI and subsequently cloned into the corresponding restriction sites of the pFastbac1 vector to produce the recombinant plasmid, pFastPVP2. The insert of the recombinant plasmid was confirmed by DNA sequencing.

After the recombinant pFastPVP2 donor plasmid was determined to be correct, the DNA was transformed into DH10Bac™ for transposition into the bacmid. The transposition assay and subsequent transfection steps were the same for all vectors. White colonies contained the recombinant bacmid, and therefore were selected for isolation of recombinant bacmid DNA. Before DNA was isolated, candidate colonies were streaked to ensure they were truly white. Bacmid DNA (B-pFastPVP2) was extracted by the phenol-chloroform extraction method. The recombinant Bacmid (B-pFastPVP2) was then analyzed by PCR.

### 2.3. Expression of the VP2 protein in sf9 cells

The recombinant baculoviruses, containing the coding sequences of VP2 with the polyhistidine tag at the N-terminus, were generated by using the Bac-to-BacTM system (Invitrogen; Luckow et al., 1993). Propagation of the recombinant virus was performed according to standard procedures (Summers et al., 2006). For production of the recombinant VP2 proteins, *sf9 *(*Spodoptera frugiperda*) cells were grown in 2-l Erlenmeyer flasks on orbital shakers (120 rpm) to a concentration of about 2 × 10^6 ^cells per ml of culture medium (30 ml growing volume) and infected with the recombinant viruses at a multiplicity of infection (MOI) of 2-3. At 72 h postinfection (p.i.), cells were collected and processed as described below. The infected cells were collected by low-speed centrifugation at 3500 × *g *(Hermle Labortechnik, Wehingen, Germany; swing-out rotor 4 × 750 ml) for 15 min at 4°C and solubilized in 30 ml of ice-cold lysis buffer [20 mM Tris, 0.3 M NaCl, 1.0% (v/v) Triton X-100, pH 7.4] for 15 min with gentle mixing (about 20 × 10^6 ^cells/ml). The crude cell lysate was clarified by high-speed centrifugation at 23,400 × *g *(Sorvall, Thermo Fisher Scientific, Waltham, MA; GSA rotor) for 20 min at 4°C. The supernatant fraction was collected, and the soluble recombinant protein products purified by IMAC as described below.

### 2.4. Purification of PPV VP2 protein

Cells were harvested at different times after infection, centrifuged at 200 × *g *for 15 min, and resuspended in 25 mM Na_2_HCO_3_, pH 8.3, at a density of 2 × 10^7 ^cells/ml; lysis was allowed to occur for 20 min. Afterward, cell debris was removed by centrifugation at 10,000 *g *for 15 min. The recombinant fusion protein VP2 was purified by IMAC. The clarified lysate was incubated with 3 ml of pre-equilibrated Ni2+- Streamline Chelating GelTM (Amersham Biosciences, Piscataway, NJ) on a rotating wheel for 16 h at 4°C, and was then placed in a 10-ml chromatography column (PolyPrep 0.8 by 4 cm; BioRad, Hercules, CA). Weakly bound and contaminating proteins were washed from the chelating gel by using 10× the column volume (20 mM Tris, 0.3 M NaCl, 20 mM imidazole, pH 7.4). The recombinant polyhistidine-tagged protein products were finally eluted from the packed bed with 3-4× the column volume (20 mM Tris, 0.3 MNaCl, 500 mM Mimidazole, pH 7.4). One-ml fractions were collected, and the protein contents were analyzed using a NanoDrop ND-1000 spectrophotometer (NanoDrop Technologies, Wilmington, DE).

### 2.5. SDS-PAGE and Western blotting

The purity and the apparent molecular weight of the recombinant VP2 specific proteins were assessed by sodium dodecyl sulfate polyacrylamide gel electrophoresis (SDS-PAGE) and immunoblot analysis. The purified proteins were separated by SDS-PAGE and were either stained with Commassie Brilliant Blue or were transferred onto nitrocellulose membranes using a wet transfer cell for Western blotting. The protein expressed 0 to 5 days after insect cells were challenged was obtained for Western blotting. The membranes were blocked with 5% skimmed milk in TBS-T (50 mM Tris-HCl, 150 mM NaCl; 0.05% Tween 20, pH 7.5) for 1 h at room temperature (RT). Swine anti-PPV sera (1:1000 dilution) or anti-His monoclonal antibody (1:5000 dilution) was added to the membranes and shaken overnight at 4°C.

The membranes were then washed three times (5 min each time) with TBS-T. Secondary antibody, either anti-swine or anti-mouse at 1:5000 dilution, was then added and incubated for 1 h. After the membranes were washed, 3,3'-diaminobenzidine (DAB) was added for colour development.

### 2.6. Hemagglutination assay

Following the method of Senda et al. [20], two-fold dilutions of samples of VP2 protein were prepared, mixed with guinea pig red blood cells, and added to the wells of a 96-well plate. After 60 minutes at 37°C, the wells were photographed.

## 3. Results

### 3.1 Construction of recombinant plasmids pFastpVP2 and recombinant bacmid

**Figure 1:**
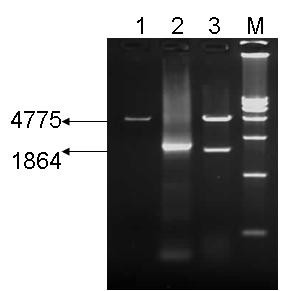
**Restriction endonuclease digests of PPV VP2 plasmid DNA**. Products produced by restriction enzyme digestion of the vector plasmid (lane 1), the PCR product of the VP2 gene (lane 2), and the recombinant plasmid (lane 3). Lane M shows the DL15000 DNA marker.

The recombinant plasmids pFastpVP2 and B-pFastpVP2 were identified by BamHI and SalI enzyme digestion. The fragments were about 1800 bp and 4775 bp, respectively, which conformed to the expected sizes (Figure 1). Recombinant bacmid was identified by PCR with the primers PPV-VP2 FD and PPV-VP2 FD. The fragment was about 1840 bp.

### 3.2 Expression and purification of polyhistidine-tagged VP2 of PPV

We have previously shown that formation of chimeric VLPs of PPV fusion constructs is feasible in pET-PPV with *E. coli *cells (Qi T et al., 2009). The recombinant baculoviruses encoding the structural proteins VP2 of PPV N-terminally fused to a polyhistidine tag (6 × his) were engineered to simplify the overall purification process of these the viral antigens. The recombinant baculoviruses were used for infection of *Sf9 *insect cells according to established procedures, and the infected insect cells were collected and solubilized by treatment with a non-ionic detergent. The cell lysates were clarified by centrifugation, and the viral proteins were finally extracted from the cytoplasmic extracts by IMAC (Ni2+). After isolation, the recombinant VP2 specific fusion proteins were analyzed by SDS-PAGE, Western-blotting, and hemagglutination assay (HA).

### 3.3 SDS-PAGE and Western blotting

**Figure 2:**
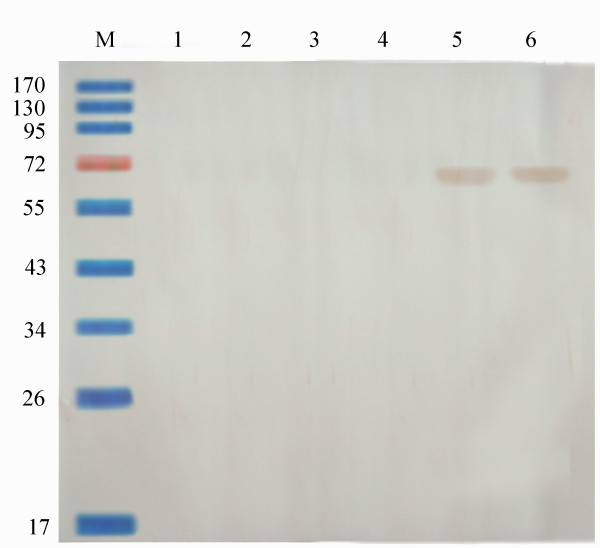
**Western blot analysis of 6-His-tagged recombinant PPV VP2 protein expressed at different times after insect cells were infection with baculovirus**. lane 1, the protein expressed in insect cells challenged with negative baculovirus; lanes 2 to 6, the protein expressed 1 to 5 days after insect cells were challenged with recombinant baculovirus; M, prestained protein ladder. The first antibody is PPV-positive pig sera, the second antibody is horseradish peroxidase-conjugated rabbit anti-pig antibody.

The VP2 protein was identified with the SDS-PAGE and Western blotting. To confirm the identity of his-tagged VP2, the purified fusion protein was subjected to Western blot assay using PPV-positive pig sera. The polycolonal antibodies recognized his-tagged VP2, and the band had the appropriate molecular weight. Immunoblot of these membranes using anti-PPV antibodies showed that the fusion protein had epitopes derived from PPV. The Western blots of purified protein obtained 0 to 5 days after insect cells were challenged indicated that the protein was expressed on day 4 and 5 (Figure 2).

**Figure 3:**
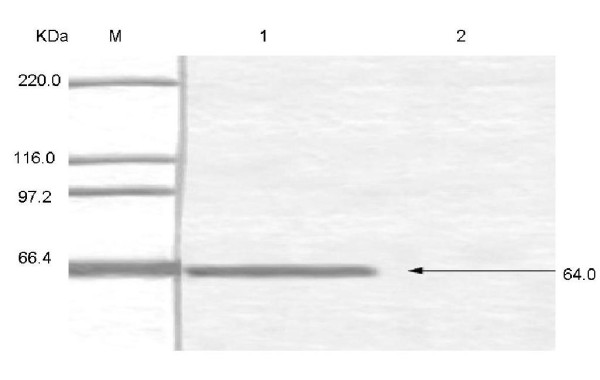
**Western blot analysis of expressed VP2**. M: prestained protein ladder, 1: the purified VP2 without the His-Tag, 1: the control of VP2 before induction. The first antibody is PPV-positive pig sera, the second antibody is horseradish peroxidase-conjugated rabbit anti-pig antibody.

To purify the fusion protein (his-tagged VP2), Ni-NTA agarose were used to finish the SDS-PAGE and Western blotting. The results showed that the target protein could be conjugated to the resin. A single band was detected by SDS-PAGE Western blotting (Figure 3).

To confirm the identity of VP2 minus the his-tag tail, the purified VP2 without a His-Tag was subjected to Western blotting assay using PPV-positive pig sera. The polyclonal antibodies recognized VP2 without a His-Tag, and the band had a molecular weight of 64 KDa. The assay therefore provided evidence that the protein could be used as an efficient immunological reagent. This conclusion was supported by the HA (see next two sections).

### 3.4 HA

**Figure 4:**
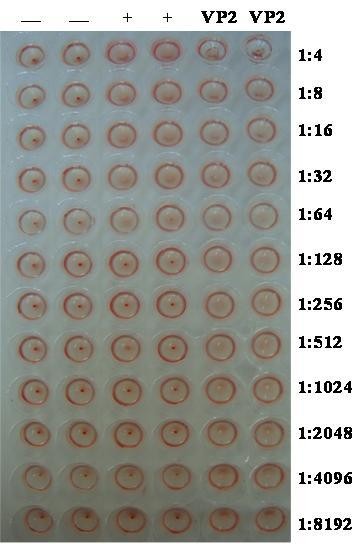
**Hemagglutination assay with the VP2 protein (two columns on right), with a sample known to be negative for PPV (two columns on the left), or with a sample known to be positive for PPV (two center columns)**.

Sample VP2 protein caused hemagglutination when diluted up to 1:8,192 (Figure 4). Therefore, the HA titer of this protein stock was 8,192.

## 4. Discussion

Currently, vaccines against PPV are produced by chemically inactivating isolated virus particles grown in primary cell cultures of porcine origin. The method is both labor intensive and costly, with the additional hazard of requiring the handling of large quantities of infectious virus [21]. Economic and safety considerations, as well as practical limitations associated with low yields of PPV particles from *in vitro *cultures, led us to the investigate recombinant sub-unit vaccines for PPV. The VP2 protein of PPV had been previously shown to self-assemble into virus-like particles when expressed in insect cells by baculovirus infection [7]. In addition, the virus-like particles of PPV were found to be highly immunogenic, and breeding sows were protected against reproductive failure in PPV challenge experiments [22]. Nonetheless, baculovirus-based systems for the production of recombinant proteins are still technically demanding, requiring sterile bioreactors that may be prohibitively costly for the production of vaccines for farm animals. Given that PPV causes serious economic losses for swine producers, development of safe, effective, and inexpensive methods for producing vaccines and diagnosing the disease is warranted.

This paper describes a method for producing the VP2 protein of PPV in an insect-baculovirus cell system. After expression was optimized, a his-tagged VP2 was obtained. The paper also describes an alternative method (IMAC) for efficient recovery of PPV VP2 and for purifying the protein using a Ni-NTA affinity column chromatography. This purification method avoids time-consuming ultracentrifugation steps such as sucrose gradient or cesium chloride gradient centrifugation. When larger amounts of recombinant proteins are needed, the purification process could be easily scaled-up by using an expanded-bed adsorption column technique. The virus-like particles formed by the fusion protein had high HA titer, could be useful as antigens for detecting PPV and could be useful for the development of a vaccine against PPV that is effective but less expensive than current vaccines [23].

The authors have previously expressed PPV VP2 in E. coli using the plasmid pET-32a (+) [17]. VP2 expressed in bacteria had similar antigenicity to native PPV VP2, as determined by Western blot analysis using polyclonal antibodies from pigs vaccinated against PPV [17]. Although PPV VP2 expressed in bacteria appears to have good immunogenicity, it is better for using as vaccine than the use as diagnosis antigen, for the antibody for E. coli in sera bothers the ELISA assay. This provide us a compelling reason for expressing PPV VP2 in a baculovirus system. The authors note a number of other studies where recombinant PPV VP2 expressed in baculovirus has been engineered with other viral antigens or immunogenic epitopes to produce multivalent vaccine candidates. The current diagnostic tests and vaccines for PPV are well established and considered to be adequate for most practical purposes; therefore the authors would like to demonstrate that any new diagnostic technology using recombinant PPV VP2 has advantages over current diagnostic tests and that any new vaccines against PPV using VP2 are superior to current vaccines in the future.

## Competing interests

The authors declare that they have no competing interests.

## Authors' contributions

GY and HZ carried out the molecular studies, and drafted the manuscript. SC participated in the design of the study and conceived of the study, and participated in its design and coordination. All authors read and approved the final manuscript.

## References

[B1] Rueda P Fominaya J Langeveld JP Bruschke C Vela C Casal JI Effect of different baculovirus inactivation procedures on the integrity and immunogenicity of porcine parvovirus-like particles Vaccine 2000 19 726 734 10.1016/S0264-410X(00)00259-0 11115693

[B2] Allan GM Kennedy S McNeilly F Foster JC Ellis JA Krakowka SJ Meehan BM Adair BM Experimental reproduction of severe wasting disease by co-infection of pigs with porcine circovirus and porcine parvovirus Pathol 1999 1 1 11 10.1053/jcpa.1998.0295 10373289

[B3] Krakowka S Ellis JA Meehan B Kennedy S McNeilly F Allan G Viral Wasting Syndrome of Swine: Experimental reproduction of postweaning multisystemic wasting syndrome in gnotobiotic swine by coinfection with Porcine Circovirus 2 and Porcine Parvovirus Vet Pathol 2000 37 254 263 10.1354/vp.37-3-254 10810990

[B4] Segales J Allan GM Domingo M Porcine circovirus diseases Anim Health Res Rev 2005 6 119 42 10.1079/AHR2005106 16583778

[B5] Gardner IA Carpenter TE Leontides L Parsons TD Financial evaluation of vaccination and testing alternatives for control of parvovirus-induced reproductive failure in swine J Am Vet Med Assoc 1996 208 863 869 8617643

[B6] Parke CR Burgess GW An economic assessment of porcine parvovirus vaccination Aust Vet J 1993 70 177 180 10.1111/j.1751-0813.1993.tb06124.x 8393655

[B7] Martinez C Dalsgaard K de Turiso JAL Cortes E Velac Casal JI Production of porcine parvovirus empty capsids with high immunogenic activity Vaccine 1992 10 10 684 90 10.1016/0264-410X(92)90090-7 1523879

[B8] Kamstrup S Langeveld J Botner A Mapping the antigenic structure of porcine parvovirus at the level of peptides Virus Res 1998 53 163 73 10.1016/S0168-1702(97)00145-7 9620208

[B9] Si Y-h Fang M-g Wang H-z [Expression of porcine parvovirus vp2 gene and construction of virus like particles (Chinese)] Virologica Sinica 2006 21 148 152

[B10] Bergeron J Hebert B Tijssen P Genome organization of the Kresse strain of porcine parvovirus: identification of the allotropic determinant and comparison with those of NADL-2 and field isolates J Virol 1996 70 4 2508 15 8642680 10.1128/jvi.70.4.2508-2515.1996 PMC190096

[B11] Li J Carroll J Ellar DJ Crystal Structure of Insecticidal d-Endotoxin from Bacillus thuringiensis at 2.5 Å resolutions Nature 1991 353 6347 815 821 10.1038/353815a0 1658659

[B12] Truyen U Parrish CR Canine and feline host ranges of canine parvovirus and feline panleukopenia virus: distinct host cell tropisms of each virus in vitro and in vivo J Virol 1992 66 9 5399 408 1323703 10.1128/jvi.66.9.5399-5408.1992 PMC289096

[B13] Pan Q He K Huang K Development of recombinant porcine parvovirus-like particles as an antigen carrier formed by the hybrid VP2 protein carrying immunoreactive epitope of porcine circovirus type 2 Vaccine 2008 26 17 2119 26 18378364 10.1016/j.vaccine.2008.02.037

[B14] Maranga L Brazao TF Carrondo MJ Virus-like particle production at low multiplicities of infection with the baculovirus insect cell system Biotechnology and Bioengineering 2003 84 245 253 10.1002/bit.10773 12966582

[B15] Luckow VA Luckow VA Lee SC Barry GF Olins PO Efficient generation of infectious recombinant baculoviruses by site-specific transposon-mediated insertion of foreign genes into a baculovirus genome propagated in Escherichia coli J Virol 1993 67 8 4566 79 8392598 10.1128/jvi.67.8.4566-4579.1993 PMC237841

[B16] Michel PO Mäkelä AR Korhonen E Toivola J Hedman L Söderlund-Venermo M Hedman K Oker-Blom CJ Purification and analysis of polyhistidine-tagged human parvovirus B19 VP1 and VP2 expressed in insect cells J Virol Methods 2008 152 1-2 1 5 10.1016/j.jviromet.2008.06.006 18598721

[B17] Qi T Cui S Expression of porcine parvovirus VP2 gene requires codon optimized Ecoli cells Virus Genes 2009 10.1007/s11262-009-0378-6 19543964

[B18] Lo-Man R Rueda P Sedlik C Deriaud E Casal I Leclerc C A recombinant virus-like particle system derived from parvovirus as an efficient antigen carrier to elicit a polarized Th1 immune response without adjuvant Eur J Immunol 1998 28 4 1401 7 10.1002/(SICI)1521-4141(199804)28:04<1401::AID-IMMU1401>3.0.CO;2-M 9565380

[B19] Hu YC Bentley WE Edwards GH Vakharia VN Chimeric infectious bursal disease virus-like particles expressed in insect cells and purified by immobilized metal affinity chromatography Biotechnol Bioeng 1999 63 6 721 9 10.1002/(SICI)1097-0290(19990620)63:6<721::AID-BIT10>3.0.CO;2-O 10397829

[B20] Senda M Hirayama N Yamamoto H Kurata K An improved hemagglutination test for study of canine parvovirus Vet Microbio 1986 12 1 1 6 10.1016/0378-1135(86)90035-0 3727364

[B21] Casal JI Use of parvovirus-like particles for vaccination andinduction of multiple immune responses Biotechnol Appl Biochem 1999 29 141 150 10075910

[B22] Casal JI Parvovirus diagnostic and vaccine production in insect cells Cytotechnology 1996 20 261 270 10.1007/BF00350405 22358489

[B23] Antonis AFG Bruschke CJM Paloma R Maranga L Casal JI Vela C Hilgers LAT Belt PBGM Weerdmeester K Carrondo MJT Langeveld JPM A novel recombinant virus-like particle vaccine for prevention of porcine parvovirus-induced reproductive failure Vaccine 2006 24 5481 5490 10.1016/j.vaccine.2006.03.089 16730104

